# Distance to a Drying Saline Lake and Lung Function Development in a Rural Border Cohort of Children

**DOI:** 10.1001/jamanetworkopen.2026.4812

**Published:** 2026-04-03

**Authors:** Fangqi Guo, Elizabeth M. Kamai, Sandrah P. Eckel, Dayane Duenas Barahona, Luis Olmedo, Esther Bejarano, Christian Torres, Christopher Zuidema, Edmund Seto, Shohreh F. Farzan, Jill E. Johnston

**Affiliations:** 1 Department of Population and Public Health Sciences, Keck School of Medicine, University of Southern California, Los Angeles; 2 Department of Environmental & Occupational Health, University of California, Irvine, Irvine; 3 Comite Civico del Valle, Brawley, California; 4 Department of Environmental & Occupational Health Sciences, University of Washington, Seattle

## Abstract

**Importance:**

The Salton Sea, a drying saline lake in southeastern California, is a growing source of wind-blown dust. Its long-term impact on children’s lung function growth remains unclear.

**Objective:**

To investigate the association of proximity to the Salton Sea and dust exposure with children’s lung function growth trajectories.

**Design, Setting, and Participants:**

For this longitudinal cohort study, community-engaged research was conducted in Imperial Valley, California, in partnership with Comité Civico del Valle, a long-standing local community organization. Children in first to third grades from 5 elementary schools near the Salton Sea were invited to participate between May 1, 2017, and May 27, 2019. Participants with missing data on core variables or with only 1 spirometry measure were excluded from analysis. Lung function assessment was conducted from March 22, 2019, to July 25, 2022; data analyses were conducted from July 2024 to July 2025.

**Exposures:**

The primary exposure was the distance from each child’s home to the edge of the Salton Sea, with residence near the sea defined as less than 11 km. Secondary exposures included particulate matter (PM) and dust storm hours (PM_10_ > 150 µg/m^3^), estimated using data from regulatory monitors.

**Main Outcomes and Measures:**

Lung function was assessed via spirometry (forced expiratory volume in 1 second [FEV_1_] and forced vital capacity [FVC]). Covariates included age, sex, ethnicity, socioeconomic status, height, body mass index, baseline respiratory health, and asthma status.

**Results:**

Of 499 children enrolled, 369 were included in the final analysis (205 [55.6%] female and 164 [44.4%] male), with a mean (SD) baseline age of 10.1 (0.6) years and mean (SD) follow-up of 2.0 (1.0) years, contributing a total of 1146 spirometry measurements (approximately 3 per participant). In fully adjusted mixed effects models, living near the sea (<11 km) was associated with 52.18 mL/y lower FVC growth (95% CI, −100.96 to −3.40 mL/y; *P* = .04), compared with living farther from the sea. The association with FEV_1_ did not reach statistical significance. More hours of dust event exposure were associated with lower FVC (β = −4.10 mL/y; 95% CI, −7.55 to −0.75 mL/y) and FEV_1_ growth (β = −2.26 mL/y; 95% CI, −4.22 to −0.29 mL/y), with a stronger association between dust event and FVC observed among children living near the sea (*P* for interaction = .04).

**Conclusions and Relevance:**

In this cohort study of school-aged children, closer residential proximity to the Salton Sea was associated with reduced lung function growth. These findings underscore the need to address environmental degradation in the region to protect children’s respiratory health.

## Key Points

**Question:**

Is living closer to the drying Salton Sea and experiencing more dust events associated with children’s lung function development over time?

**Findings:**

In a cohort study of 369 school-aged children tracked over 2 years, those living within 11 km of the Salton Sea had significantly reduced growth in forced vital capacity compared with children living farther from the sea. Increased dust exposure was also associated with reduced lung function growth, particularly among children living near the sea.

**Meaning:**

These findings suggest that environmental exposures near the drying Salton Sea may impair lung function development in children.

## Teaser

This cohort study examines whether proximity to a drying saline lake in southeastern California and related dust exposure are associated with children’s lung function growth trajectories.

## Introduction

Drought and extreme heat, together with human activities, have contributed to significant water declines in many inland lakes worldwide. The drying of these saline water bodies, such as Lake Chad, the Aral Sea, and Owens Lake, has resulted in large areas of exposed dry lakebeds, also known as playas.^1,2,3,4^ These playas are significant sources of wind-blown dust and may result in dramatic increases in local particulate matter (PM) levels.^5^ While common challenges exist with shrinking lakes, each lake has unique characteristics that influence dust generation and environmental impacts.^6^ Therefore, investigating each case individually is crucial for developing context-specific solutions.^6,7^

The Salton Sea is a 375-square-mile, drying terminal saline lake in Imperial County, California, near the US and Mexico border.^5,8^ Located 235 feet below sea level in one of the state’s driest regions, it was formed in 1905 after a breach in a Colorado River irrigation canal and has been sustained for over a century primarily by agricultural runoff.^5,8^ Studies project that approximately 40% of the lakebed will be exposed by 2030, leading to an 11% rise in PM_10_ emissions across the region.^9^ The residents near the Salton Sea are predominantly a rural, marginalized population (>80% Hispanic or Latino) facing high rates of unemployment and poverty.^8^

Earlier research has shown that exposure to increased PM levels from the Salton Sea is linked to increased wheeze and other respiratory symptoms in local children.^10,11^ Nonetheless, the long-term impact of the shrinking Salton Sea on children’s lung function development remains unclear. Lung function, assessed by spirometry, typically undergoes a growth spurt in adolescence, peaks in young adulthood, and gradually declines thereafter.^12^ Early-life lung function growth may predict peak lung function in young adulthood and later respiratory disease risk. The immature immune system in childhood may be particularly sensitive to environmental exposures.^13,14^ However, little is known about how exposure to drying saline lakes may influence children’s lung function trajectories.

This community-engaged research was conducted in collaboration with Comité Civico del Valle,^8^ a long-standing community organization in Imperial Valley.^8^ Comité Civico del Valle facilitated partnerships with local schools and community members and contributed to cohort planning and research question development. Through these efforts, we developed the Children’s Assessing Imperial Valley Respiratory Health and the Environment (AIRE) Study to investigate how environmental exposures, particularly from the Salton Sea, influence children’s respiratory health.^8^ The aims of the current study were to investigate (1) the association of proximity to the Salton Sea with children’s lung function growth trajectories and (2) whether proximity to the sea modifies the association between PM exposures and children’s lung function growth.

## Methods

### Data

The AIRE prospective cohort study recruited children in first to third grade from 5 elementary schools in Imperial Valley, along with their parents, between May 1, 2017, and May 27, 2019; participants contributed spirometry data from March 22, 2019, to July 25, 2022. Participants were excluded from analysis if they were missing core variables, including date of birth, height, or body mass index (BMI). Because this study assessed change in lung function over time, participants with a single spirometry measurement were also excluded

We conducted health assessments at 5 elementary schools, coordinating with classroom schedules to obtain measurements during the school day. Data were obtained using bilingual health questionnaires and in-person examinations, capturing information on demographics, lifestyle, medical history, respiratory symptoms, anthropometric measures, and spirometry. Questionnaires were adapted from the International Study of Asthma and Allergies in Childhood toolbox^15^ and the Southern California Children’s Health Study.^16^

Incentives were provided at each data collection wave, including art supplies or classroom gift cards for teachers, coffee or gas gift cards for parents, and small prizes for children. Written consent was obtained from all parents before their children’s participation. This study was reviewed and approved by the University of Southern California’s institutional review board. Our analysis follows the Strengthening the Reporting of Observational Studies in Epidemiology (STROBE) reporting guideline.

### Lung Function Assessment

As the children reached the fourth grade (approximately 9 to 10 years old), we assessed lung function using a commercially available Easy-On PC spirometer (ndd Medical Technologies). Trained staff administered maximal-effort spirometry tests in accordance with American Thoracic Society guidelines. To ensure consistent and acceptable measurements, each child performed between 3 and 7 maneuvers.^17^ The spirometer software recorded multiple variables, including forced expiratory volume in forced vital capacity (FVC), the first second of exhalation (FEV_1_), and maximal midexpiratory flow (MMEF). For our analysis, we focused on FVC and FEV_1_, as both are established, independent predictors of respiratory disease, cardiovascular mortality, and all-cause mortality.^18^ Since children’s FVC and FEV_1_ growth from age 9 to 13 years follows a roughly linear trend (eFigure 1 in Supplement 1), we examined linear lung function growth during the study period as the main outcome.

### Distance to the Sea and PM Exposure

We calculated the distance between the child’s home and the edge of the Salton Sea on a yearly basis, and a mean distance to the Salton Sea variable was generated throughout the study period. For dichotomized distance, participants living less than 11 km from the edge of the Salton Sea were considered near, and those living at least 11 km from the sea were considered far away (eFigure 2 in Supplement 1).

To estimate participants’ PM exposure and dust event hours in this rural area, we used a nearest-monitor approach.^19,20^ Ambient air quality data for PM_2.5_ and PM_10_ were obtained from the 12 monitors (eFigure 2 in Supplement 1) within the California Air Resources Board (CARB) Air Quality Monitoring Information System.^21^ Participants’ caregivers were asked to provide their residential address and residential history at each study visit. If residential addresses were unavailable (4.7% of observations), the participant’s school address was used instead. For each participant, we identified the nearest CARB monitor to evaluate hourly PM levels 1 year prior to each lung function assessment.^10^ The distance from each participant’s address to the nearest PM monitor ranged from less than 100 m to 19 km, with a median distance of 2.2 km. Ninety-five percent of the participants lived within 10 km of a PM monitor. With these data, we calculated each participant’s 1-year mean concentrations of PM_2.5_ and PM_10_, as well as the 1-year total number of dust event hours (defined as the number of hours when hourly PM_10_ concentrations exceeded 150 µg/m^3^).^10^ We then calculated the mean of these annual values across all study years for each participant to obtain a single, participant-level estimate of mean exposure over the entire study period.

### Covariates

Covariates included age at each spirometry measure; time-varying height and height squared; time-varying body mass index (BMI) and BMI squared; presence of respiratory disease symptoms, treatment for respiratory disease, and presence of allergies within 3 months prior to each spirometry measurement; and staff member who administered spirometry examinations, as well as time-constant factors including sex, health insurance type (public, private, or none) as a proxy for social economic status, baseline asthma status, parents’ preferred language (Spanish or English), secondhand smoke exposure in the home, prenatal maternal smoking, and gas cooking stove at home. The distribution of these characteristics, as well as self-reported race and ethnicity, is shown in Table 1.

**Table 1.** Characteristics of the Total Study Sample and Subsamples Stratified by Distance to the Salton Sea

Characteristic	No. (%)		
	Total sample (N = 369)	Near sea (<11 km) (n = 193)	Far from sea (≥11 km) (n = 176)
Total measures, No.	1146	665	481
Baseline age, mean (SD), y	10.1 (0.6)	10.1 (0.6)	10.1 (0.6)
Follow-up length, mean (SD), y	2.0 (1.0)	2.0 (1.0)	2.0 (1.1)
Sex			
Female	205 (55.6)	107 (55.4)	97 (55.1)
Male	164 (44.4)	86 (44.6)	78 (44.3)
Race and ethnicity			
Hispanic	313 (84.8)	168 (87.1)	145 (82.4)
Non-Hispanic other^a^	4 (1.1)	1 (0.5)	3 (1.7)
Non-Hispanic White	22 (6.0)	10 (5.2)	12 (6.8)
Not reported	30 (8.1)	14 (7.3)	16 (9.1)
Caregiver’s preferred language			
English	235 (63.7)	123 (63.7)	112 (63.6)
Spanish	134 (36.3)	70 (36.3)	64 (36.4)
Baseline height, mean (SD), cm	140.7 (7.5)	140.6 (7.6)	140.9 (1.3)
Baseline BMI, mean (SD), kg/m^2^	21.2 (5.3)	21.2 (5.5)	21.3 (5.0)
Health insurance type			
None	27 (7.3)	17 (8.8)	10 (5.7)
Private	98 (26.6)	50 (25.9)	48 (27.3)
Public	211 (57.2)	113 (58.5)	98 (55.7)
Not reported	33 (8.9)	13 (6.7)	20 (11.4)
Baseline asthma			
No	276 (74.8)	141 (73.1)	135 (76.7)
Yes	93 (25.2)	52 (26.9)	41 (23.3)
Air pollution exposure, mean (SD)^b^			
PM_2.5_, µg/m^3^	7.9 (1.5)	6.8 (0.8)	9.0 (1.3)
PM_10_, µg/m^3^	41.5 (4.6)	40.2 (5.0)	42.9 (3.8)
Dust event hours	211.7 (57.0)	219.1 (42.8)	203.6 (68.5)
Spirometry measures, mean (SD)			
Baseline FVC, mL	2283.6 (612.4)	2237.2 (617.5)	2334.6 (604.5)
Baseline FEV_1_, mL	1910.5 (408.0)	1884.7 (437.1)	1938.7 (372.7)
FVC growth, mL/y^c^	316.4 (13.7)	283.0 (17.1)	357.0 (21.2)
FEV_1_ growth, mL/y^c^	245.7 (10.6)	223.0 (13.0)	273.4 (16.9)

Abbreviations: BMI, body mass index; FEV_1_, forced expiratory volume in 1 second; FVC, forced vital capacity; PM, particulate matter.

^a^ Non-Hispanic other includes participants who identified as African American or Pacific Islander. Race and ethnicity are self-reported.

^b^ Air pollution exposures were the mean across the study period.

^c^ Crude FVC and FEV_1_ growth were estimated using linear mixed-effects models that included a participant-level random intercept, with age as a fixed-effect variable. Lung function growth stratified by distance from the sea was estimated by adding an interaction term between age and distance from the sea to the model. Reported values are the estimated fixed-effect coefficients of age ± the corresponding standard errors of the estimation.

These variables were selected a priori based on existing research^22,23^ and evaluations conducted in our model (eTable 1 in Supplement 1). Most covariates (eg, demographics and health status) were collected using self-administered questionnaires completed by the caregivers. Race and ethnicity were collected because participants were recruited from a county with a predominantly Hispanic population. Height and weight were objectively measured 3 times by staff at each study visit.

### Statistical Analysis

Data analyses were conducted from July 2024 to July 2025. We summarized categorical variables using frequencies and percentages, and continuous variables using means and SDs. Distributions were also stratified by residential distance to the Salton Sea (<11 km vs ≥11 km). The cutoff was based on the sample median of 10.6 km.

We first employed linear mixed-effects models to assess the association of distance to the sea with FVC and FEV_1_ growth, adjusting for the covariates described above. Models included school-level and participant-level random intercepts to account for clustering and repeated measurements. Age was centered at the baseline mean (approximately 10 years). The interaction term between age and distance to the sea was a key term in the model, which examines whether the rate of lung function growth (ie, the slope over time) differed by distance to the sea. Distance to the sea was investigated as both continuous and dichotomous variables. Additionally, dust event hours and PM levels were used as secondary exposures. The CIs presented were calculated using robust standard error.^24^

We also investigated whether the association of PM exposure, asthma status, and sex with lung function growth varied by the distance to the sea. To test this, we added separate 3-way interaction terms to the models (distance to sea × age × each variable of interest). Results displayed interaction *P* values and coefficients and CIs for each subgroup. *P* < .05 indicated statistical significance. All analyses were done using STATA SE, version 17.0 (StataCorp LLC).

## Results

A total of 731 children were recruited, of whom 499 contributed spirometry data. Of these, 9 participants were excluded due to missing core variables. An additional 122 with a single spirometry measurement were also excluded, resulting in a final analytic sample size of 369.

Table 1 displays the characteristics of the total sample (n = 369). Participants had a mean (SD) baseline age of 10.1 (0.6) years and were followed for a mean (SD) of 2.0 (1.0) years, contributing a total of 1146 spirometry measurements (approximately 3 per participant). Our study sample has slightly more females than males (205 [55.6%] vs 164 [44.4%]). Most participants identified as Hispanic (313 [84.8%]), while 22 (6.0%) identified as non-Hispanic White, 4 (1.1%) identified as non-Hispanic other (including African American or Pacific Islander), and 30 (8.1%) did not report race or ethnicity. More than half of study participants (211 [57.2%]) relied on public health insurance and 27 (7.3%) were uninsured. Asthma was present in 93 (25.2%) of the study participants at enrollment.

Among children living near the sea (<11 km) and far from the sea (≥11 km), the median (IQR) distances to the sea were 9.7 (9.0-10.1) km and 21.5 (20.5-37.1) km, respectively. Children living near vs farther from the sea were comparable in demographics, social economic status, and baseline health (Table 1). Air pollution exposures varied by distance to the sea, with children living near the sea having more hours of dust events yet slightly lower mean ambient concentrations of PM_2.5_ and PM_10_ as compared with children living farther from the sea.

**Figure 1.**
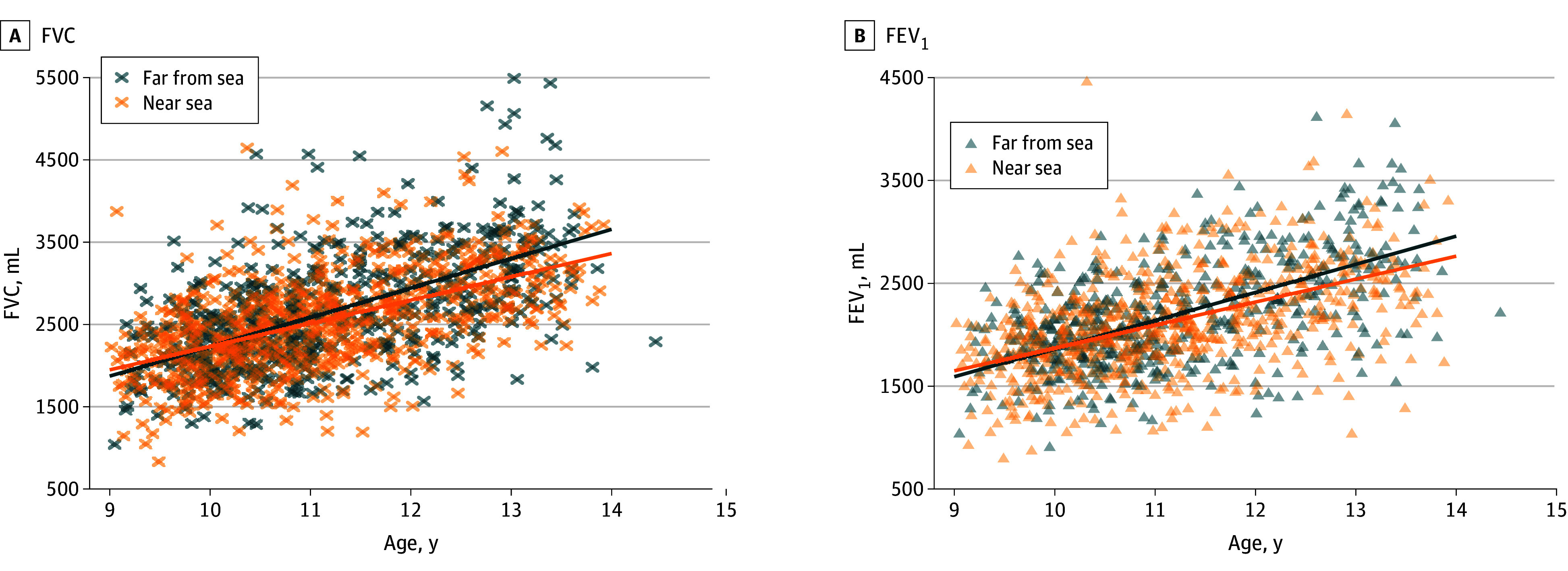
Scatterplot of Development of Lung Function Over Time by Distance to the Salton Sea Crude associations between age and lung function among children living near the sea (<11 km) and farther from the sea (≥11 km). Fitted lines were generated from single-exposure multilevel mixed-effects models that included age, distance to the sea, and their interaction, and included participant-level random intercept. FEV_1_ indicates forced expiratory volume in 1 second; FVC, forced vital capacity.

Among the entire sample, crude FVC and FEV_1_ growth measured a mean (SD) of 316.4 (13.7) mL/y and 245.7 (10.6) mL/y, respectively, during the follow-up period. Children living near the sea showed lower crude FVC and FEV_1_ growth compared with those living far from the sea (Table 1, Figure 1). In fully adjusted models (Table 2), we observed that each additional kilometer closer to the sea that a participant lived was associated with a 2.69 mL/y decrease in FVC growth (95% CI, −4.75 to −0.63 mL/y; *P* = .01. When distance to the sea was analyzed as a dichotomous variable, children living near the sea showed slower lung function growth by −52.18 mL/y in FVC (95% CI, −100.96 to −3.40 mL/y; *P* = .04) compared with children living farther away from the sea. The difference in FEV_1_ by distance to the sea showed a similar trend but did not reach statistical significance. Baseline FVC and FEV_1_ did not differ significantly between the 2 groups (eTable 2 in Supplement 1).

**Table 2.** Adjusted Associations of Annual Lung Function Growth With Distance to the Salton Sea and Dust Events^a^

Lung function metric and exposure^b^	β (95% CI)^c^	*P* value
**FVC growth, mL/y**		
Distance, per km closer to sea	−2.69 (−4.75 to −0.63)	.01
Near sea, vs reference of ≥11 km	−52.18 (−100.96 to −3.40)	.04
PM_10_ Dust event hours, per 10-h increase	−4.10 (−7.45 to −0.75)	.02
**FEV_1_ growth, mL/y**		
Distance, per km closer to sea	−2.00 (−4.12 to 0.12)	.07
Near sea, vs reference of ≥11 km	−38.70 (−82.96 to 5.55)	.09
PM_10_ Dust event hours, per 10-h increase	−2.26 (−4.22 to −0.29)	.02

Abbreviations: FEV_1_, forced expiratory volume in 1 second; FVC, forced vital capacity; PM, particulate matter.

^a^ Results obtained from single-exposure multilevel mixed-effects models with school-level and participant-level random intercepts. Estimated associations with growth slopes were obtained from the coefficient of interaction between age and exposure. Models were adjusted for time-varying covariates (age; height; height squared; body mass index [BMI]; BMI squared; presence of respiratory disease symptoms, treatment for respiratory disease, and presence of allergies within 3 months prior to each spirometry measurement; and spirometry staff member), as well as time-constant covariates (sex, health insurance type, baseline asthma status, parents’ preferred language [Spanish or English], secondhand smoke exposure in the home, prenatal maternal smoking, and gas cooking stove at home).

^b^ Exposure metrics were continuous (distance or dust event hours) with effect estimates scaled to the unit change listed (ie, difference in annual lung function growth per 1 kilometer closer to the sea or 10-hour increase in PM_10_ dust event exposure) or binary (near vs far from sea) with effect estimates interpreted as difference in annual lung function growth comparing participants living near the sea (<11 km) with those living far from the sea (≥11 km).

^c^ Growth slope β estimates were obtained from the fixed-effect interaction coefficients between age and each variable of interest.

Dust event exposure was also associated with reduced lung function growth (Table 2). Every additional 10 hours of dust event exposure was associated with a 4.10 mL/y decrease in FVC growth (95% CI, −7.55 to −0.75 mL/y; *P* = .02) and a 2.26 mL/y decrease in FEV_1_ growth (95% CI, −4.22 to −0.29 mL/y; *P* = .02). PM_10_ and PM_2.5_ levels were not significantly associated with FEV_1_ or FVC growth (eTable 3 in Supplement 1).

**Figure 2.**
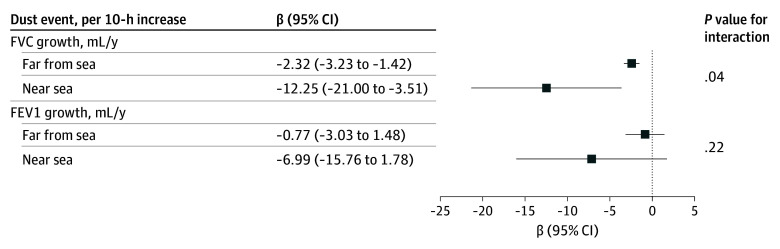
Dot Plot of Adjusted Associations of Annual Lung Function Growth With Dust Events, Stratified by Residential Distance to the Sea Coefficients and 95% CIs for the association between dust event hours and lung function growth in the overall sample, children living near the sea (<11 km), and children living far from the sea (≥11 km). Results were obtained from single-exposure, multilevel mixed-effects models that included school-level and participant-level random intercepts. Models were adjusted for time-varying covariates (age; height; height squared; body mass index [BMI]; BMI squared; presence of respiratory disease symptoms, treatment for respiratory disease, and presence of allergies within 3 months prior to each spirometry measurement; and spirometry staff member) as well as time-constant covariates (sex, health insurance type, baseline asthma status, parents’ preferred language [Spanish or English], secondhand smoke exposure in the home, prenatal maternal smoking, and gas cooking stove at home). A 3-way interaction term (distance to sea × age × dust event hours) was added to models to obtain stratified estimates.

When stratifying the sample by distance to the sea (Figure 2), we observed a consistent pattern that the association between dust events and FVC growth was stronger among children living near the sea, compared with those living farther away. For example, among children living farther away from the sea, every additional 10 hours of dust event exposure was associated with a 2.32 mL/y decrease in FVC growth (95% CI, −3.22 to −1.42 mL/y); while among those living near the sea, FVC growth was reduced by 12.25 mL/y (95% CI, −21.00 to −3.51 mL/y; *P* for interaction = .04).

**Figure 3.**
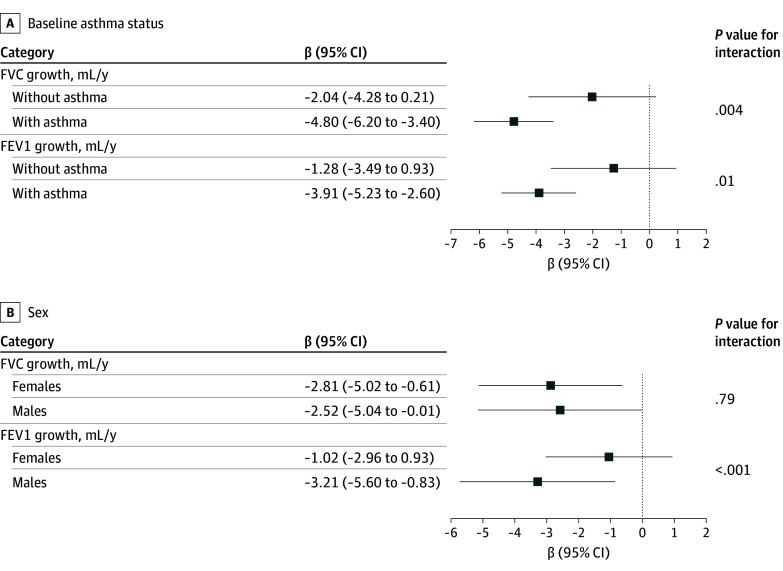
Dot Plot of Adjusted Associations of Distance to the Sea With Lung Function Growth, Stratified by Baseline Asthma Status and Sex Coefficients and 95% CIs for the association between distance to the sea and lung function growth stratified by baseline asthma status (A) and sex (B). Results were obtained from single-exposure multilevel mixed-effects models that included school-level and participant-level random intercepts. Models were adjusted for time-varying covariates (age; height; height squared; body mass index [BMI]; BMI squared; presence of respiratory disease symptoms, treatment for respiratory disease, and presence of allergies within 3 months prior to each spirometry measurement; and spirometry staff member, as well as time-constant covariates (sex, health insurance type, baseline asthma status, parents’ preferred language [Spanish or English], secondhand smoke exposure in the home, prenatal maternal smoking, and gas cooking stove at home). A 3-way interaction term (distance to sea × age × asthma or sex) was added to models to obtain stratified estimates.

Children with asthma generally exhibited slower FVC or FEV_1_ growth compared with those without asthma (eTable 1 in Supplement 1). In subgroup analyses by asthma status, we found that the association of distance to the sea with FVC and FEV_1_ growth was of greater magnitude among children with baseline asthma, compared with those without asthma (Figure 3). For each additional kilometer closer to the sea, asthmatic children exhibited significant decreases in both FVC and FEV_1_: −4.80 mL/y (95% CI, −6.20 to −3.40 mL/y) and −3.91 mL/y (95% CI, −5.23 to −2.60 mL/y), respectively; while among children without asthma, distance to sea was not significantly associated with lung function. Additionally, while distance to the sea had a similar association with FVC growth in both males and females, distance was more strongly associated with FEV_1_ in males (β = −3.21; 95% CI, −5.60 to −0.83) than in females (β = −1.02; 95% CI, −2.96 to 0.93).

## Discussion

In this longitudinal cohort study of elementary school–aged children, we found that living closer to the Salton Sea was associated with deficits in lung function development. Over a mean of 2 years of follow-up in a cohort of children, we found that measures of typical lung function growth, as characterized by increases in both FVC and FEV_1_, were smaller for those living within 11 km of the Salton Sea compared with those living farther away. Notably, the magnitude of this association with lung function growth was comparable to that reported for children living within 500 m of a freeway.^23^

Over the past 2 decades, more extreme weather and human activities, such as water transfers and conservation measures, have contributed to reduced water inflows to the Salton Sea and the exposure of approximately 16 000 new acres of dry lakebed.^25,26^ This has contributed to elevated PM_10_ levels and increased health risks for nearby children.^10,11^ Asthma prevalence in communities around the sea exceeds the California average.^11^ Elevated PM levels and dust events,^27,28^ partially due to the drying Salton Sea, have been associated with worse lung function and increased wheeze and bronchitis symptoms, especially among children living closer to the Salton Sea (<11 km).^10^ However, prior studies were cross-sectional, and the longitudinal research on the lake’s long-term health effects is lacking.^29^

Our trajectory analysis expands our understanding of the Salton Sea’s influence on pediatric respiratory health by showing that proximity to the sea is associated with reduced lung function growth in children from age 10 through 12 years. Our findings are consistent with a cross-sectional study conducted around another drying saline lake, in which Kunii et al^30^ found that children aged 6 to 15 years living closer to the Aral Sea in central Asia had a higher prevalence of cough and wheezing, as well as lower FVC, compared with those who lived farther away. Notably, we did not observe significant differences in lung function by distance to the sea at baseline (eTable 1 in Supplement 1), suggesting that the period from age 10 to 12 years, possibly due to accelerated development of the lungs during this time, may represent a critical window of vulnerability to environmental exposures. However, examining this question will require critical window analyses with longer follow-up periods.

In addition to proximity to the sea, ambient dust events were also associated with lung function growth. We found that children exposed to more hours of PM_10_ dust events showed greater deficits in FVC growth, although PM_10_ or PM_2.5_ concentrations were not significantly associated with lung function growth across all participants. Similar findings have been reported in previous cohort studies in urban areas. For example, the Southern California Children’s Health Study, based in urban Los Angeles, reported that exposure to ambient PM levels as well as traffic from highways were associated with deficits in growth of lung function from age 10 to 18 years.^22,23^ Our study adds to this body of research by showing that PM_10_ dust exposure is also significantly associated lung function growth in this rural cohort of children, specifically among children living closer to the sea.

The exact components of PM around the Salton Sea remain under investigation, but adsorbed sulfate, chloride, pesticides, and toxic metals are likely key components of PM_10_.^29^ These differ from the PM_10_ components that have been reported in urban areas, where vehicular emissions are the primary source. Typically, exposure to PM impacts respiratory health through mechanisms such as oxidative stress and inflammatory injury in the lower respiratory tract.^31^ However, some evidence from experimental models suggests that dust from the Salton Sea may trigger a distinct inflammatory response, characterized by innate immune responses, which differs from a classic allergic response.^32^ Further studies are needed to characterize the specific dust components that may be related to these patterns of inflammation and to examine whether they translate to human populations.

### Strengths and Limitations

Our study has several strengths. First, we are among the first environmental health studies to investigate the impact of a drying saline lake on children’s lung function growth over time. Additionally, we objectively measured children’s lung function (FVC and FEV_1_) using spirometry and also accounted for the episodic nature of dust events in rural communities when assessing exposures. Our findings provided important insight into how proximity to the Salton Sea and local PM levels are associated with children’s FVC and FEV_1_ growth.

This study also has some limitations. First, we used regulatory PM monitor values assigned to residential addresses to estimate PM concentrations, which may not fully capture individual exposure levels. Second, the average follow-up period was from age 10 to 12 years, which coincides with a typical lung function growth spurt in childhood.^33^ Patterns of lung function growth during this period may differ from lung growth during other life stages. Third, study participants are a subset of children who had more than 1 spirometry measure. However, we compared the demographics of our study sample with the full AIRE cohort, and no significant differences were observed.

## Conclusions

In this cohort study, we found that proximity to the Salton Sea was negatively associated with children’s lung function growth in a rural cohort of children aged 10 to 12 years. Our study expanded the limited understanding of how drying lakes influence population-level pediatric health over time.^34^ The drying Salton Sea has been a critical environmental issue for decades. Although the California Natural Resources Agency has made efforts to address this issue, current actions are insufficient to reduce its environmental influence on local children’s health.^29^ There is an urgent need for a more comprehensive approach to dust management as well as stronger community involvement in decision-making to improve pediatric health.

## Notes

Supplement 1. **eTable 1.** Crude associations between each covariate and lung function growth: results of univariate mixed-effects models **eTable 2.** Adjusted associations of distance to the Salton Sea with baseline spirometry measurements (n=369) **eTable 3.** Adjusted associations of PM levels with lung function growth (milliliter per year) **eFigure 1.** Distribution of FVC and FEV1 by age (n=369, total measures=1146) **eFigure 2.** Particulate matter monitors and count of observations of AIRE cohort participants (2017–2019) by census tract

Supplement 2.
Data Sharing Statement

## References

[1] Gao H, Bohn TJ, Podest E, McDonald KC, Lettenmaier DP. On the causes of the shrinking of Lake Chad. Environ Res Lett. 2011;6(3):034021. doi:10.1088/1748-9326/6/3/034021

[2] Bennion P, Hubbard R, O’Hara S, ; Médecins san Frontières/Aral Sea Respiratory Dust and Disease project team. The impact of airborne dust on respiratory health in children living in the Aral Sea region. Int J Epidemiol. 2007;36(5):1103-1110. doi:10.1093/ije/dym195 17911152

[3] Cowley JM, Deering-Rice CE, Lamb JG, . Pro-inflammatory effects of inhaled Great Salt Lake dust particles. Part Fibre Toxicol. 2025;22(1):2. doi:10.1186/s12989-025-00618-9 39819386 PMC11737234

[4] Gillette D, Ono D, Richmond K. A combined modeling and measurement technique for estimating windblown dust emissions at Owens (dry) Lake, California. J Geophys Res Earth Surf. 2004;109(F1). doi:10.1029/2003JF000025

[5] Tompson AFB. Born from a flood: the Salton Sea and its story of survival. J Earth Sci. 2016;27(1):89-97. doi:10.1007/s12583-016-0630-7

[6] Dickey H, Schreuder M, Schmid B, Yimam YT. Quantifying dust emission potential of playa and desert surfaces in the Salton Sea Air Basin, California, United States. Aeolian Res. 2023;60:100850. doi:10.1016/j.aeolia.2022.100850

[7] Jones BA, Fleck J. Shrinking lakes, air pollution, and human health: Evidence from California’s Salton Sea. Sci Total Environ. 2020;712:136490. doi:10.1016/j.scitotenv.2019.136490 31931219

[8] Farzan SF, Kamai E, Duenas Barahona D, . Cohort profile: the Assessing Imperial Valley Respiratory Health and the Environment (AIRE) study. Paediatr Perinat Epidemiol. 2024;38(4):359-369. doi:10.1111/ppe.13065 38450855 PMC11116055

[9] Parajuli SP, Zender CS. Projected changes in dust emissions and regional air quality due to the shrinking Salton Sea. Aeolian Res. 2018;33:82-92. doi:10.1016/j.aeolia.2018.05.004

[10] Johnston JE, Kamai E, Duenas Barahona D, . Air quality and wheeze symptoms in a rural children’s cohort near a drying saline lake. Environ Res. 2024;263(Pt 2):120070. doi:10.1016/j.envres.2024.120070 39406285 PMC11609001

[11] Farzan SF, Razafy M, Eckel SP, Olmedo L, Bejarano E, Johnston JE. Assessment of respiratory health symptoms and asthma in children near a drying saline lake. Int J Environ Res Public Health. 2019;16(20):3828. doi:10.3390/ijerph16203828 31614424 PMC6843482

[12] Jordan BK, McEvoy CT. Trajectories of lung function in infants and children: setting a course for lifelong lung health. Pediatrics. 2020;146(4):e20200417. doi:10.1542/peds.2020-0417 32938776 PMC7546086

[13] US Environmental Protection Agency. Children are not little adults! February 12, 2014. Accessed November 27, 2024. https://www.epa.gov/children/children-are-not-little-adults

[14] Dunea D, Iordache S, Pohoata A. fine particulate matter in urban environments: a trigger of respiratory symptoms in sensitive children. Int J Environ Res Public Health. 2016;13(12):12. doi:10.3390/ijerph13121246 27983715 PMC5201387

[15] Asher MI, Keil U, Anderson HR, . International Study of Asthma and Allergies in Childhood (ISAAC): rationale and methods. Eur Respir J. 1995;8(3):483-491. doi:10.1183/09031936.95.08030483 7789502

[16] Peters JM, Avol E, Navidi W, . A study of twelve Southern California communities with differing levels and types of air pollution. I. Prevalence of respiratory morbidity. Am J Respir Crit Care Med. 1999;159(3):760-767. doi:10.1164/ajrccm.159.3.9804143 10051248

[17] Graham BL, Steenbruggen I, Miller MR, . Standardization of Spirometry 2019 Update. An Official American Thoracic Society and European Respiratory Society Technical Statement. Am J Respir Crit Care Med. 2019;200(8):e70-e88. doi:10.1164/rccm.201908-1590ST 31613151 PMC6794117

[18] Rydell A, Janson C, Lisspers K, Lin YT, Ärnlöv J. FEV_1_ and FVC as robust risk factors for cardiovascular disease and mortality: insights from a large population study. Respir Med. 2024;227:107614. doi:10.1016/j.rmed.2024.107614 38670319

[19] Berhane K, Zhang Y, Salam MT, . Longitudinal effects of air pollution on exhaled nitric oxide: the Children’s Health Study. Occup Environ Med. 2014;71(7):507-513. doi:10.1136/oemed-2013-101874 24696513 PMC4310696

[20] Zhang JS, Gui ZH, Zou ZY, . Long-term exposure to ambient air pollution and metabolic syndrome in children and adolescents: a national cross-sectional study in China. Environ Int. 2021;148:106383. doi:10.1016/j.envint.2021.106383 33465664

[21] California Air Resources Board. Air Quality and Meteorological Information System. Updated March 12, 2020. Accessed January 14, 2025. https://www.arb.ca.gov/aqmis2/aqmis2.php

[22] Gauderman WJ, Avol E, Gilliland F, . The effect of air pollution on lung development from 10 to 18 years of age. N Engl J Med. 2004;351(11):1057-1067. doi:10.1056/NEJMoa040610 15356303

[23] Gauderman WJ, Vora H, McConnell R, . Effect of exposure to traffic on lung development from 10 to 18 years of age: a cohort study. Lancet. 2007;369(9561):571-577. doi:10.1016/S0140-6736(07)60037-3 17307103

[24] Freedman DA. On the so-called “Huber Sandwich Estimator” and “Robust Standard Errors.” Am Stat. 2006;60(4):299-302. doi:10.1198/000313006X152207

[25] Sapozhnikova Y, Bawardi O, Schlenk D. Pesticides and PCBs in sediments and fish from the Salton Sea, California, USA. Chemosphere. 2004;55(6):797-809. doi:10.1016/j.chemosphere.2003.12.009 15041284

[26] Xu EG, Bui C, Lamerdin C, Schlenk D. Spatial and temporal assessment of environmental contaminants in water, sediments and fish of the Salton Sea and its two primary tributaries, California, USA, from 2002 to 2012. Sci Total Environ. 2016;559:130-140. doi:10.1016/j.scitotenv.2016.03.144 27058132

[27] Guo F, Kamai EM, Eckel SP, . Dust events and children’s lung function near a drying saline lake. Am J Respir Crit Care Med. 2025;211(11):2133-2136. doi:10.1164/rccm.202504-0799RL 41072400

[28] Guo F, Kamai EM, Eckel SP, . Particulate matter levels and children’s lung function in a rural cohort near a drying saline lake. Environ Epidemiol. 2026;10(1):e455. doi:10.1097/EE9.0000000000000455 41522885 PMC12788894

[29] Johnston JE, Razafy M, Lugo H, Olmedo L, Farzan SF. The disappearing Salton Sea: a critical reflection on the emerging environmental threat of disappearing saline lakes and potential impacts on children’s health. Sci Total Environ. 2019;663:804-817. doi:10.1016/j.scitotenv.2019.01.365 30738261 PMC7232737

[30] Kunii O, Hashizume M, Chiba M, . Respiratory symptoms and pulmonary function among school-age children in the Aral Sea region. Arch Environ Health. 2003;58(11):676-682. doi:10.3200/AEOH.58.11.676-682 15702891

[31] Ghio AJ, Kummarapurugu ST, Tong H, . Biological effects of desert dust in respiratory epithelial cells and a murine model. Inhal Toxicol. 2014;26(5):299-309. doi:10.3109/08958378.2014.888109 24669951

[32] Biddle TA, Yisrael K, Drover R, . Aerosolized aqueous dust extracts collected near a drying lake trigger acute neutrophilic pulmonary inflammation reminiscent of microbial innate immune ligands. Sci Total Environ. 2023;858(Pt 3):159882. doi:10.1016/j.scitotenv.2022.159882 36334668

[33] Wang X, Dockery DW, Wypij D, Fay ME, Ferris BG Jr. Pulmonary function between 6 and 18 years of age. Pediatr Pulmonol. 1993;15(2):75-88. doi:10.1002/ppul.1950150204 8474788

[34] Hendryx M, Fedorko E, Halverson J. Pollution sources and mortality rates across rural-urban areas in the United States. J Rural Health. 2010;26(4):383-391. doi:10.1111/j.1748-0361.2010.00305.x 21029174

