# Supplementary material for: Distance to a Drying Saline Lake and Lung Function Development in a Rural Border Cohort of Children
Source: JAMA Netw Open. 2026 Apr 3;9(4):e264812. doi: 10.1001/jamanetworkopen.2026.4812 (PMC13049490; doi:10.1001/jamanetworkopen.2026.4812)
Supplement: Supplement 1. — eTable 1. Crude associations between each covariate and lung function growth: results of univariate mixed-effects models eTable 2. Adjusted associations of distance to the Salton Sea with baseline spirometry measurements (n=369) eTable 3. Adjusted associations of PM levels with lung function growth (milliliter per year) eFigure 1. Distribution of FVC and FEV1 by age (n=369, total measures=1146) eFigure 2. Particulate matter monitors and count of observations of AIRE cohort participants (2017–2019) by census tract [file jamanetwopen-e264812-s001.pdf]

Supplemental Online Content

Guo F, Kamai EM, Eckel SP, et al. Distance to a drying saline lake and lung function development in a rural border cohort of children. *JAMA Netw Open*. 2025;9(4):e264812. doi:10.1001/jamanetworkopen.2026.4812

eTable 1. Crude associations between each covariate and lung function growth: results of univariate mixed-effects models

eTable 2. Adjusted associations of distance to the Salton Sea with baseline spirometry measurements (n=369)

eTable 3. Adjusted associations of PM levels with lung function growth (milliliter per year)

eFigure 1. Distribution of FVC and FEV1 by age (n=369, total measures=1146)

eFigure 2. Particulate matter monitors and count of observations of AIRE cohort participants (2017–2019) by census tract

This supplemental material has been provided by the authors to give readers additional information about their work.

© 2026 Guo F et al. JAMA Network Open.

eTable 1. Crude associations between each covariate and lung function growth: results of univariate mixed-effects models.

			FVC growth (mL/year)				FEV1 growth (mL/per year)			
	n	n	Beta	95% CI		p	Beta	95% CI		p
Height (cm)	369	1146	4.8	2.3	7.2	<0.00	2.6	0.6	4.6	0.012
Height squared	369	1146	0.0	0.0	0.0	0.003	0.0	0.0	0.0	0.112
BMI (kg/m^2)	369	1146	1.6	-2.5	5.6	0.448	-0.7	-4.1	2.6	0.674
BMI squared	369	1146	0.0	-0.1	0.1	0.947	0.0	-0.1	0.0	0.377
Sex										
Female	205	642	Ref.							
Male	164	504	63.6	10.9	116.3	0.018	33.1	-8.7	74.8	0.120
Ethnicity										
Non-Hispanic	26	83	Ref.							
Hispanic	313	981	-79.1	-171.5	13.2	0.093	-35.4	-129.0	58.2	0.459
Not reported	30	82	-105.1	-216.3	6.0	0.064	-67.0	-188.1	54.1	0.278
Language										
English	235	712	Ref.							
Spanish	134	434	-35.3	-90.2	19.7	0.208	2.8	-40.2	45.8	0.899
Insurance type										
None	27	78	Ref.							
Private or Mexicali	98	307	59.6	-43.3	162.5	0.256	25.1	-69.0	119.2	0.601
Public	211	667	-16.5	-113.2	80.2	0.738	-8.3	-99.7	83.1	0.859
Not reported	33	94	-33.4	-143.3	76.4	0.551	-40.4	-155.3	74.5	0.491
Baseline asthma status										
No	276	851	Ref.							
Yes	93	295	-58.7	-113.9	-3.5	0.037	-73.3	-117.3	-29.2	0.001
Respiratory disease symptom										
No	304	980	Ref.							
Yes	65	166	-77.5	-150.4	-4.7	0.037	-59.3	-123.5	4.9	0.070
Treatment										
No	352	1088	Ref.							
Yes	17	58	-78.0	-185.6	29.5	0.155	23.8	-81.2	128.9	0.657
Allergies										
No	308	967	Ref.							
Yes	61	179	-1.8	-77.5	73.9	0.963	31.3	-31.3	93.9	0.327
Gas cooking stove at home										
No	82	253	Ref.							
Yes	255	804	43.5	-30.5	117.6	0.249	28.3	-25.0	81.7	0.298
Not reported	32	89	-0.5	-94.8	93.7	0.991	-9.0	-98.7	80.8	0.845
Prenatal mother smoking										
No	312	975	Ref.							
Yes	26	81	11.4	-88.3	111.1	0.823	-55.3	-141.5	31.0	0.209

© 2026 Guo F et al. JAMA Network Open.

Not reported	31	90	-17.2	-88.8	54.4	0.638	-23.9	-99.6	51.8	0.536
Baseline secondhand smoking										
No	298	920	Ref.							
Yes	28	111	49.6	-22.5	121.7	0.178	40.2	-42.9	123.4	0.343
Not reported	43	115	34.9	-86.7	156.5	0.574	7.5	-72.3	87.3	0.853
School										
1	125	441	Ref.							
2	25	92	73.1	-7.0	153.1	0.074	101.1	16.8	185.3	0.019
3	69	165	129.1	53.5	204.7	0.001	87.5	26.2	148.9	0.005
4	75	231	85.9	24.8	147.0	0.006	71.5	24.1	118.8	0.003
5	75	217	118.8	37.4	200.2	0.004	105.2	49.1	161.4	<0.001

© 2026 Guo F et al. JAMA Network Open.

eTable 2. Adjusted associations of distance to the Salton Sea with baseline spirometry measurements (n=369)

	Beta	95% CI	p value
**Baseline FVC (mL)**			
Distance, per km closer to Sea	-1.5	-4.5, 1.5	0.334
Near Sea, vs. ≥ 11km (reference)	-20.3	-135.1, 94.4	0.728
**Baseline FEV1 (mL)**			
Distance, per km closer to Sea	-1.2	-4.1, 1.8	0.438
Near Sea, vs. ≥ 11km (reference)	0.3	-93.9, 94.5	0.995

Results were obtained from single-exposure multi-level mixed effects linear models that included school-level and participant-level random intercepts. Models adjusted for baseline age, height, height^2, body mass index (BMI), BMI^2, and presence of respiratory disease symptoms and treatment for respiratory disease and presence of allergies within 3 months prior to spirometry measurement, spirometry staff member, sex, health insurance type, baseline asthma status, parents preferred language (Spanish or English), secondhand smoke exposure in the home, prenatal maternal smoking, and gas cooking stove at home.

© 2026 Guo F et al. JAMA Network Open.

eTable 3. Adjusted associations of PM levels with lung function growth (milliliter per year)

	Beta[c]	95% CI	p value
**FVC growth (mL/year)**			
$PM_{2.5}$ [a]	16.7	-1.2, 34.8	0.068
PM_{10} [b]	33.7	-39.4, 106.9	0.366
**FEV1 growth (mL/year)**			
$PM_{2.5}$ [a]	13.5	-4.8, 31.8	0.147
PM_{10} [b]	34.9	-42.7, 112.6	0.378

Results were obtained from single-exposure multi-level mixed effects models that included school-level and participant-level random intercepts. Models adjusted for time-varying covariates: age, height, $height^2$, body mass index (BMI), BMI^2, and presence of respiratory disease symptoms and treatment for respiratory disease and presence of allergies within 3 months prior to each spirometry measurement, and spirometry staff member, as well as time constant covariates: sex, health insurance type, baseline asthma status, parents preferred language (Spanish or English), secondhand smoke exposure in the home, prenatal maternal smoking, and gas cooking stove at home.

[a] The coefficient is interpreted as the change in annual lung function growth per 1 μg/m^3 increase in annual $PM_{2.5}$ exposure

[b] The coefficient is interpreted as the change in annual lung function growth per 10 μg/m^3 increase in annual PM_{10} exposure

[c] Growth slope beta estimates were obtained from the coefficient of interaction between age and each variable of interest

© 2026 Guo F et al. JAMA Network Open.

eFigure 1. Distribution of FVC and FEV1 by age (n=369, total measures=1146)

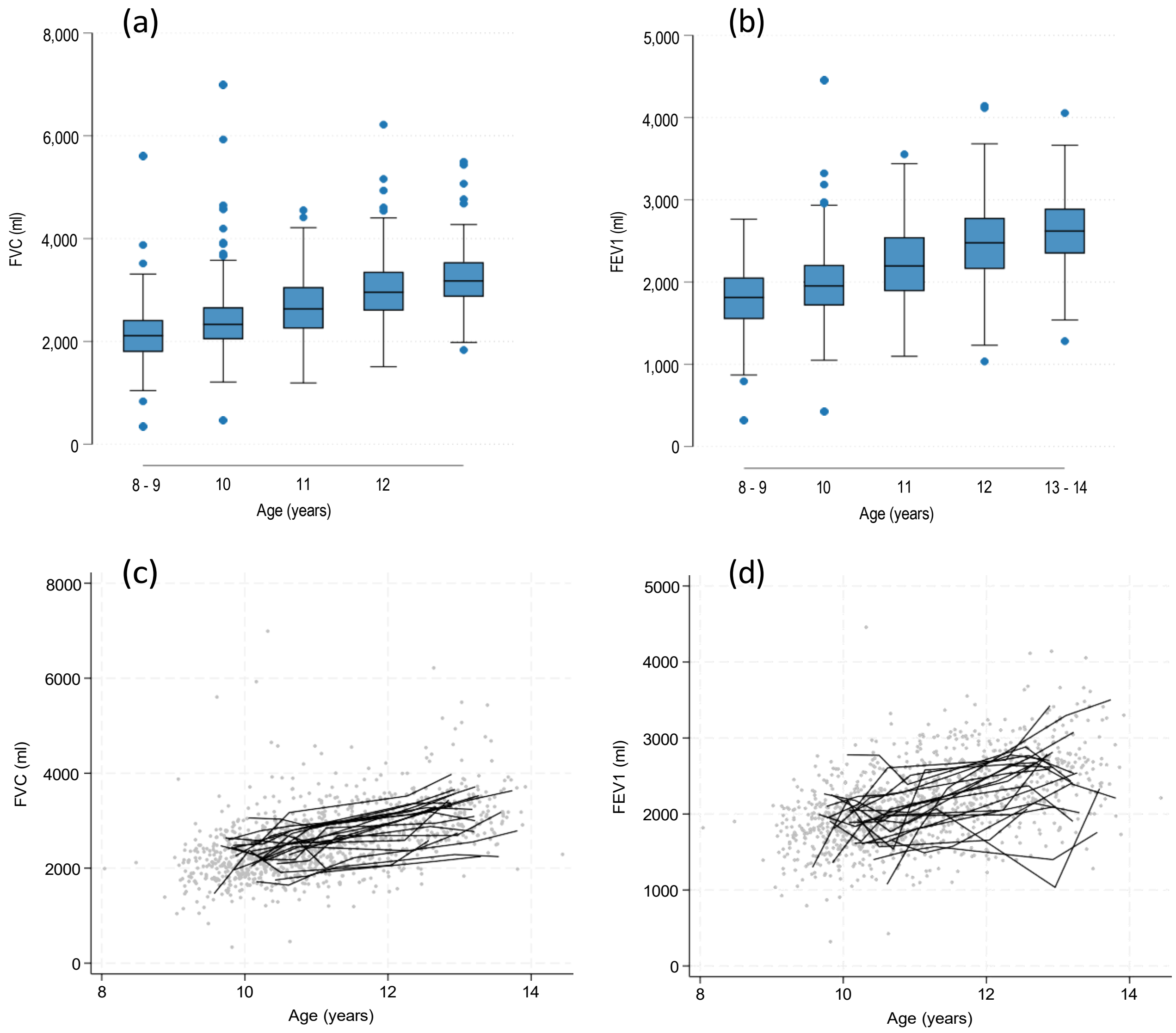

(a), (b) Boxplots of FVC and FEV1 values by age based on 1146 spirometry measurements. (c), (d) With-in participant FVC and FEV1 trend for 20 participants who contributed the greatest number spirometry measurements (5 each).

© 2026 Guo F et al. JAMA Network Open.

eFigure 2. Particulate matter monitors and count of observations of AIRE cohort participants (2017–2019) by census tract.

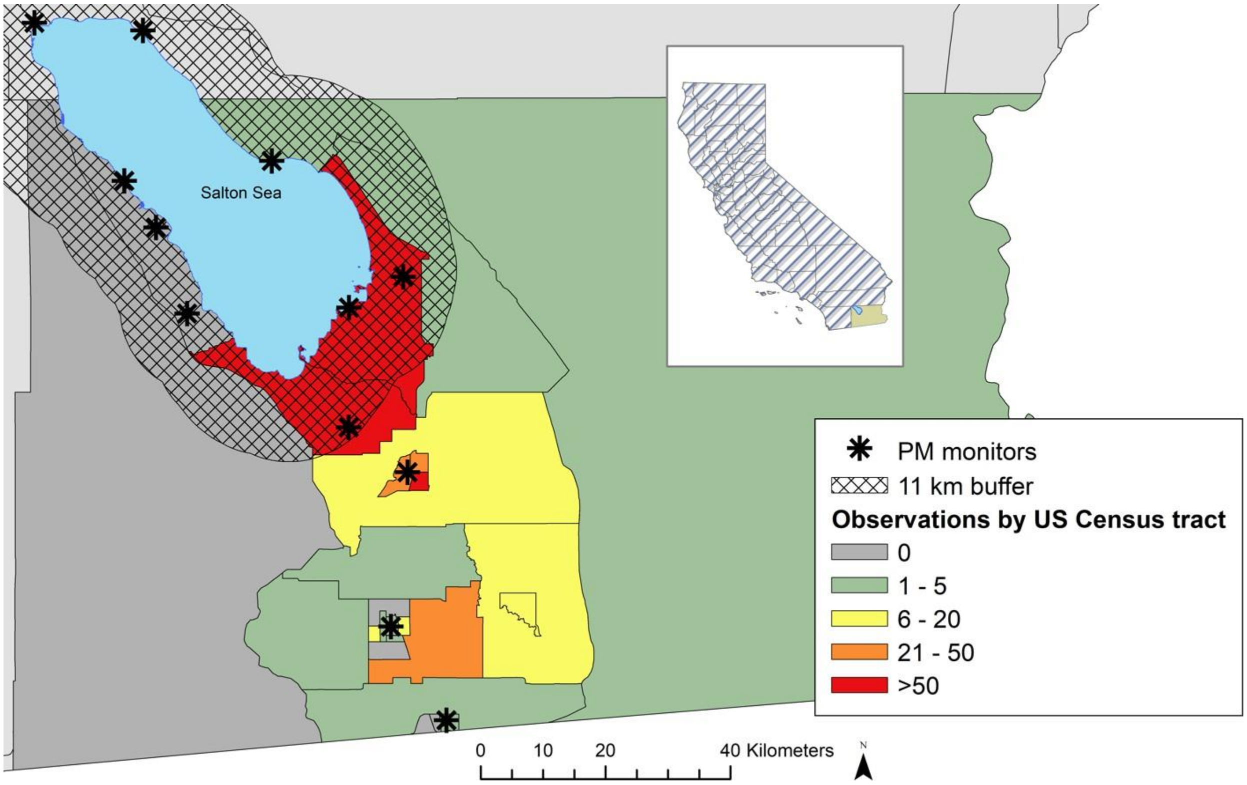

Note. This figure is adapted with permission from Johnston, J. E., et al. (2024). Environmental Research, 263, 120070. Copyright © 2024 Elsevier.

© 2026 Guo F et al. JAMA Network Open.
