# Supplementary material for: Distance to a Drying Saline Lake and Lung Function Development in a Rural Border Cohort of Children
Source: JAMA Netw Open. 2026 Apr 3;9(4):e264812. doi: 10.1001/jamanetworkopen.2026.4812 (PMC13049490; doi:10.1001/jamanetworkopen.2026.4812)
Supplement: Supplement 2. — Data Sharing Statement [file jamanetwopen-e264812-s002.pdf]

Data Sharing Statement

Guo. Distance to a Drying Saline Lake and Lung Function Development in a Rural Border Cohort of Children. *JAMA Netw Open*. Published April 03, 2026.
doi:10.1001/jamanetworkopen.2026.4812

Data
Data available: Yes
Data types: Data dictionary
How to access data: Investigators seeking access to the study data will be required to submit a research protocol to the PIs, Dr. Shohreh Farzan (sffarzan@usc.edu) and Dr. Jill Johnston (Jillj1@uci.edu), for review. If the study is deemed scientifically valid, we will provide the requested data pending assurances that the confidentiality of our study subjects will not be violated.
When available: With publication

Supporting Documents
Document types: None

Additional Information
Who can access the data: Investigators seeking access to the study data will be required to submit a research protocol to the PIs, Dr. Shohreh Farzan (sffarzan@usc.edu) and Dr. Jill Johnston (Jillj1@uci.edu), for review.
Types of analyses: If the study is deemed scientifically valid, we will provide the requested data pending assurances that the confidentiality of our study subjects will not be violated.
Mechanisms of data availability: Any data provided will be stripped of specific subject identifiers, including name, address, geographic coordinates, work locations, dates, and any other data that could be used to identify a specific participant or his/her residence. Recipients must agree to security policies, including password-protected access and encrypted storage that will minimize the risk of unauthorized distribution.
